# Multi-Metals CaMgAl Metal-Organic Framework as CaO-based Sorbent to Achieve Highly CO_2_ Capture Capacity and Cyclic Performance

**DOI:** 10.3390/ma13102220

**Published:** 2020-05-12

**Authors:** Szu-Chen Wu, Po-Hsueh Chang, Chieh-Yen Lin, Cheng-Hsiung Peng

**Affiliations:** 1 Department of Materials Science and Engineering, National Chiao Tung University, 1001 University Road, Hsinchu 300, Taiwan; s320431@hotmail.com (S.-C.W.); iggy1002@gmail.com (C.-Y.L.); 2 Department of Chemical and Materials Engineering, Ming Hsin University of Science and Technology, 1 Xinxing Road, Hsin-Feng Hsinchu 304, Taiwan; pohsueh.chang@gmail.com

**Keywords:** CaMgAl-MOF, CaO, carbon dioxide (CO_2_) capture

## Abstract

In this study, Ca-based multi-metals metal-organic framework (CaMgAl-MOF) has been designed as precursor material for carbon dioxide (CO_2_) capture to enhance the CO_2_ capture capacity and stability during multiple carbonation-calcination cycles. The CaMgAl-MOFs were constructed from self-assembly of metal ions and organic ligands through hydrothermal process to make metal ions uniformly distributed through the whole structure. Upon heat treatment at 600 °C, the Ca-based multi-metals CaMgAl-MOF would gradually transform to CaO and MgO nanoparticles along with the amorphous aluminum oxide distributed in the CaO matrix. XRD, Fourier transform infrared (FTIR), and SEM were used to identify the structure and characterize the morphology. The CO_2_ capture capacity and multiple carbonation-calcination cyclic tests of calcined Ca-based metal-organic framework (MOF) (attached with O and indicated as Ca-MOF-O) were performed by thermal gravimetric analysis (TGA). The single metal component calcined Ca-MOF sorbent have the highest CO_2_ capture capacity up to 72 wt.%, but a lower stability of 61% due to severe particle aggregation. In contrast, a higher Ca-rich MOF oxide sorbent with tailoring the Mg/Al ratios, Ca_0.97_Mg_0.025_Al_0.005_-MOF-O, showed the best performance, not only having the high stability of ~97%, but also maintaining the highest capacity of 71 wt.%. The concept of using Ca-based MOF materials combined with mixed-metal ions for CO_2_ capture showed a potential route for achieving efficient multiple carbonation-calcination CO_2_ cycles.

## 1. Introduction

Carbon dioxide (CO_2_) accumulation in atmosphere has been believed to be one of the main reasons for global warming [1,2]. Pathways for CO_2_ emission mitigation are being developed, including the improvements of energy efficiency and carbon capture and storage (CCS) in order to limit this temperature increase in 2 °C. The implementation of the CCS technique to current power plants and industries is considered to be the most practical solution to the greenhouse gases (GHGs) problem. The main strategies of high-temperature CO_2_ adsorption/separation have been focused on using inorganic solid sorbents, including calcium oxides [3,4,5,6,7,8,9,10]. However, most of the commonly studied adsorbents suffer from low capacity at elevated temperatures, because the CO_2_ adsorption capacity of these materials decreases drastically with increasing temperature. Feasible improvements are being adopted by incorporating inert support material to act as a physical barrier to CaO particles, but the capture efficiency and capacity of CO_2_ adsorption would be reduced [11,12,13,14].

**Scheme 1:**
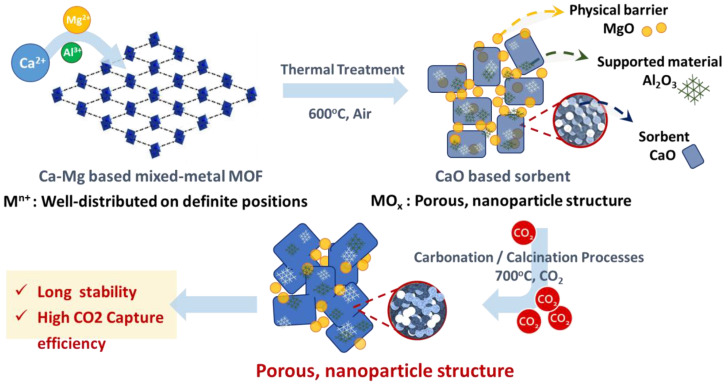
Mechanism of calcined porous multi-metal-ions Ca-based multi-metals metal-organic framework (CaMgAl-MOF) with a high CO_2_ capture efficiency and long stable cyclic performance.

In contrast, metal-organic frameworks (MOFs) are well recognized for their extraordinary surface areas and ultrahigh porosity applied for energy storage and environmental remediation [15,16]. The pore size and functionality of MOFs can be tailored by rational selection of the organic ligand, functional group, and metal ion. Several strategies have been adopted to improve the performance of MOFs in CO_2_ capture applications at low temperatures during the past several years [17,18]. However, little progress has been achieved to tune the MOF structures for enhancing CO_2_ uptake at intermediate-high temperatures. MOFs can be used as self-sacrificing template through calcination to generate the high-temperature sorbent and, meanwhile, create some microporous to enhance the adsorption and escape of CO_2_ from the structure. More importantly, using MOFs as a CO_2_ capture sorbent, anti-sintering material, such as Mg, Al components can be well distributed through the synthesis of mixed-metal MOF structure, which would effectively promote the CO_2_ capture performances, as shown in Scheme 1. In this study, porous multi-metals Ca-based metal-organic frameworks (Ca-MOFs), such as Ca-based multi-metals metal-organic frameworks (CaMgAl-MOFs), were synthesized by substituting different amounts of Ca^2+^ for Mg^2+^ and Al^3+^ in the reaction solution to act as the precursor to generate CaO-based sorbent for high temperature CO_2_ capture in order to enhance both CO_2_ capacity and long-term stability during the multiple carbonation-calcination processes. The Ca-MOFs can be used as self-sacrificing templates through calcination to create porous structure, as the organic ligands were decomposed to small molecules and escaped from the oxides. In addition to creating a highly porous microporous structure, using MOFs as a CO_2_ capture media is to take advantage of the well-distributed anti-sintering material, such as Mg, Al components through the MOF structure, which would effectively promote the CO_2_ capture performances as shown in Scheme 1. The results indicated that partial Al substitution for Mg in the Ca-rich CaMg-MOF displayed significant enhancement on the CO_2_ capture with a high CO_2_ capture capacity approximately to pure CaO and maintained long-term stable carbonation-calcination cyclic performance. To our best knowledge, this is the first report to demonstrate that multi-metal-ions CaMgAl-MOF can present a much higher CO_2_ capture capacity and excellent cyclic carbonation-calcination performance.

## 2. Experiment

### 2.1. Materials

Aluminum nitrate nonahydrate (Al(NO_3_)_3_·9H_2_O, 98%), Calcium nitrate tetrahydrate (Ca(NO_3_)_2_·4H_2_O, 99%) were purchased from Sigma Aldrich (St. Louis, MO, USA). Magnesium nitrate hexahydrate (Mg(NO_3_)_2_·6H_2_O, 98%, Fluka (Charlotte, NC, USA)), N,N-dimethylacetamide (C_4_H_9_NO, DMA, 99.5%, J.T.Baker (Delaware, PA, USA)). Terephthalic acid (H_2_BDC, 98%, Alfa Aesar (Ward Hill, MA, USA)). All of the reagents were used as received. Deionized (DI) water was supplied by an in-house system.

### 2.2. Hydrothermal Synthesis of CaMgAl Metal-Organic Framework Nanocrystals

The precursor of CaO-based sorbents, CaMgAl-MOFs, were synthesized through a hydrothermal method with various amounts of Al substituted into the CaMg-MOF structure. First, 6 mmole of H_2_BDC were dissolved in a pre-heated 40 mL DMA solution at 50 °C. After complete dissolution, a colorless solution was formed; a total amount of 6 mmole metal nitrates was then added into the container. After stirring for 10–15 min., the obtained colorless reaction solution was transferred into Teflon-lined stainless-steel 100 mL autoclave and then treated at 120 °C for 18 hour. White crystalline powders were washed with ethanol through centrifugation four times, and the collected solid was dried in a 60 °C oven 24 hour. The MOF-based materials were ground into fine powder by hand grinding through an agate mortar for 1–2 min. because of slight particle aggregation before use. Different reaction time, temperature, and relative metal ratio were synthesized while using the same procedure. The as-synthesized CaMg-MOFs were denoted as Ca_x_Mg_1−x_-MOF, with x representing the Ca mole% of total metal component. On the other hand, the Ca-Mg-Al MOFs were labeled as Ca_x_Mg_y_Al_1−x−y_-MOF, as shown in Table 1, where x, y, z represented the mole% of calcium, magnesium, and aluminum element, respectively. The raw powders were then calcined at 600 °C to convert MOF precursors to form CaO-based oxide sorbents, which was attached by “-O” and denoted as Ca_x_Mg_1−x_-MOF-**O** or Ca_x_Mg_y_Al_1−x−y_-MOF-**O**.

### 2.3. Characterization

XRD analysis of the crystallization and structures of powder samples was carried out on a Bruker D2 Advance X-ray Diffractometer (Bruker, Billerica, MA, USA) in reflection mode, while using Cu λ_Kα_ = 1.5418 Å radiation. Field Emission Scanning Electron Microscope (FE-SEM) (JEOL, Tokyo, Japan) and Energy Dispersive X-ray Spectroscopy (EDXS) were performed on a JEOL JSM-6700F instrument (Abingdon, UK) with a field emitter as the electron source. The Fourier transform infrared (FTIR) measurements were collected on a PerkinElmer Spectrum 100 FTIR spectrometer (PerkinElmer, Waltham, MA, USA) while using the self-supporting KBr pallets in the wave number range from 450 to 4000 cm^−1^. Inductively Coupled Plasma-Mass Spectrometer (ICP-MS) measurements were collected on Jarrell-Ash, ICAP-9000 (Thermo Scientific, Waltham, MA, USA). The samples were digested with nitro-hydrochloric acid in order to determine Ca/Mg/Al metal ratio in as-synthesized powders. X-ray Photoelectron Spectroscopy (XPS) (Thermo Scientific, Waltham, MA, USA) measurements were carried out using spherical sector analyzer and Multi-channeltron. The source was monochromatic Al Kα source (1486.6 eV, 200 W) with a spot size of 650–120 µm. The binding energy scale of the system was calibrated using Au 4f_7/2_ 84.0 eV from freshly evaporated gold sample. Solid-state ^27^Al NMR studies were performed on BRUKER AVIII 600 WB spectrometer (Bruker, Billerica, MA, USA) with a 14.1 Tesla magnetic field, in which these nuclei resonate at 600 MHz.

### 2.4. CO_2_ Capture Capacity

Thermal gravimetric analysis (TGA) measurements were performed on a TA Instrument Q500 (TA Instrument, New Castle, DE, USA). Typically 5–8 mg powders were loaded into a platinum hanging plate to perform the tests. For the thermal property test, the samples were heated at a rate of 5 °C min^−1^ from room temperature up to 800 °C under continuous nitrogen gas flow. For the carbon dioxide multiple carbonation-clacination cyclic tests, the carbonation process was carried out at 700 °C for 25 min. in an atmosphere of carbon dioxide (N_2_ balance, 40 mL min^−1^), while the clacination process was performed in nitrogen gas flow at 700 °C for 8 min. In this research, TGA were used to quantify the CO_2_ capture capacity and stability of the sorbents according to the following Equations (1) and (2):

(1) $$\mathrm{Capacity}=\left({W-W}_{0}\right)\times 100\%$$

(2) $$\mathrm{Stability}=[1-\left({W-W}_{f}\right)/W]\times 100\%$$

where W is the weight of the sorbent after CO_2_ adsorption, W_0_ is the initial weight of the sorbent, and W_f_ is the weight of the sorbent after multiple carbonation-calcination cycles.

## 3. Results and Discussion

### 3.1. Character of MOF with Different Ca/Mg Ratios

**Figure 1:**
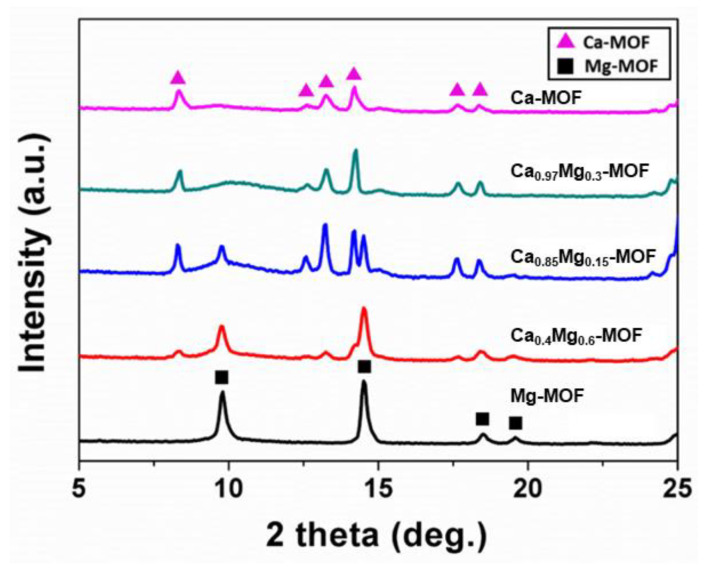
Powder X-ray diffraction showing the characteristic diffraction peaks of CaMg-MOFs.

The powder X-ray diffraction results presented in Figure 1 showed that the characteristic diffraction peaks of CaMg-MOFs were in the range between 5–25°. The characteristic diffraction 2θ peaks of pure calcium-MOF (denoted as Ca-MOF) were at 8.5°, 12.7°, 13.3°, 14.3°, 17.8°, and 18.4° (JCPDF No.46-1873). For pure magnesium-MOF, denoted as Mg-MOF, the characteristic diffraction 2θ peaks were at 9.8°, 14.5°, 18.5°, and 19.6°. When partial calcium was used to substitute for Mg, Ca/Mg mixed metal MOF could still be successfully synthesized via the same procedure. The PXRD patterns showed that with increasing Ca/Mg, the peak intensity of characteristic diffraction peaks of Ca-MOF phase increased, while those of Mg-MOF phase decreased and became amorphous, such as Ca_0.97_Mg_0.03_-MOF. Besides, as increasing Ca content in Ca_x_Mg-MOF (where x indicated relative mole ratio of Ca in Ca + Mg = 1), it was found that the peaks of Mg-MOF phase were also slightly shifted to smaller 2 theta, but peaks of Ca-MOF was slightly shifted to a higher 2 theta, indicating that both metal ions of Ca and Mg in the MOF structure may be partially accommodated in the MOF structure to cause the changes in d-spacing. Because CaMg-MOF was calcined at 600 °C, it would be completely decomposed and transformed to metal oxides, represented as CaMg-MOF-O, composed of calcium oxide, calcium carbonate (CaCO_3_), and/or magnesium oxide (MgO).

**Figure 2:**
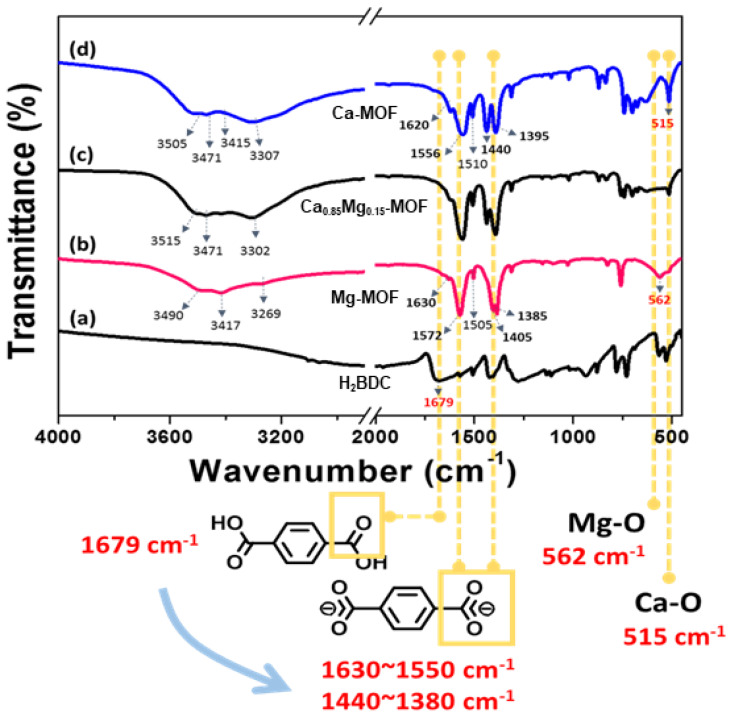
Fourier transform infrared (FTIR) spectra were used to analyze the adsorption peaks of (**a**) H_2_BDC, (**b**) Mg-MOF, (**c**) Ca_0.85_Mg_0.15_-MOF, and (**d**) Ca-MOF where the peaks at 562 cm^−1^ and 515 cm^−1^ can be assigned to Mg-O bond and the Ca-O bond, respectively, to indicate the formation of Mg-MOF and Ca-MOF.

FTIR were used to analyze the adsorption peaks of the organic functional group to assure the bonding between organic ligands and metal ions, as shown in Figure 2. During the synthesis process of CaMg-MOFs, the COOH in H_2_BDC will dissolve first, producing COO^−^ to bond with dissolved metal ions Ca^2+^ and Mg^2+^ in the solution for forming M-O bonds. The adsorption peak at 1679 cm^−1^ represented the characteristic adsorption of C=O bonds of COOH in H_2_BDC, as shown in Figure 2a. In contrast, for all of the Ca_x_Mg_1-x_-MOFs, the C=O peak disappeared while strong C-O bands at 1600–1550 cm^−1^ and 1450–1380 cm^−1^ were presented, which can be assigned to the asymmetry and symmetry stretching modes of C-O bonds in coordinated COO^−^ groups, respectively. The peak at 1505–1510 cm^−1^ was assigned to the vibration of the benzene ring. The above results indicated the coordination between COO^−^ of BDC and metal ions in MOF structure, and they were consistent with literatures [19,20]. Moreover, in Mg-MOF (Figure 2b), the peak at 562 cm^−1^ can be assigned to Mg-O bond was observed. On the other hand, the peak at 515 cm^−1^ indicating the Ca-O bond was detected in in Ca_0.85_Mg_0.15_-MOFs (Figure 2c) and Ca-MOF structure (Figure 2d). These characteristic peaks of both were all presented, confirming the formation of both Mg-MOF and Ca-MOF structures. In addition, the broad band at 3471 cm^−1^ indicated the adsorption of surface water on the Ca-MOF and Ca_0.85_Mg_0.15_-MOFs. The two extensive bands at 3515 cm^−1^ and 3302 cm^−1^ can be assigned to the asymmetric and symmetric stretching of coordinated water molecules, respectively. The same vibration and stretching band for water absorption at 3490 cm^−1^ and 3269 cm^−1^ in Mg-MOF was also observed and a slight red shift at 3417 cm^−1^ for coordinated water molecules indicated a lower bond strength in Mg-OH [21].

**Figure 3:**
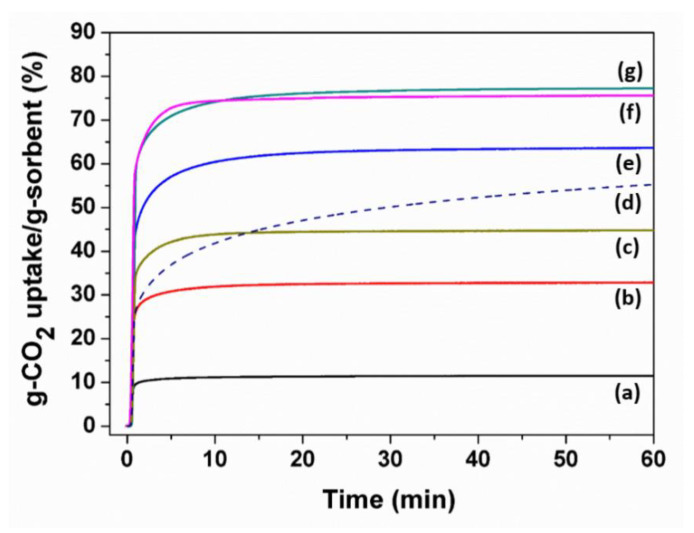
CO_2_ capture capacity at 700 °C for (**a**) Ca_0.3_Mg_0.7_-MOF-O, (**b**) Ca_0.6_Mg_0.4_-MOF-O, (**c**) Ca_0.75_Mg_0.25_-MOF-O, (**d**) Commercial CaO, (**e**) Ca_0.85_Mg_0.15_-MOF-O, (**f**) Ca_0.97_Mg_0.03_-MOF-O, and (**g**) Ca-MOF-O.

Figure 3 presents the carbonation curves of calcined MOF, designated as CaMg-MOF-O, samples with different metal ratios, indicating that all of the calcined MOF samples displayed a relatively rapid absorption of CO_2_ to reach absorption saturation in 10 min. as compared to commercial CaO nanoparticle. The single metal component Ca-MOF-O sorbent displayed the highest CO_2_ capture capacity of about 75 wt.%, which was very close to the theoretical capacity of pure CaO of 78 wt.%. The Ca_0.97_Mg_0.03_-MOF-O showed the highest capacity of 72.7 wt.%, while the Ca_0.3_Mg_0.7_-MOF-O showed the lowest capacity of 10.1 wt.% and Ca_0.85_Mg_0.15_-MOF-O sorbent with 60.6 wt.% in between. These results indicated that CaO-based MOF absorbents have a positive effect on both capture capacity and carbonation kinetics when compared to commercial CaO in Figure 3d, which displayed a slow adsorption kinetics of CO_2_ capture as compared to other Ca-Mg-MOF adsorbents. Furthermore, a rapid cycle degradation was also observed for the commercial CaO, because it produced strong aggregation and particle growth during multiple carbonation-calcination cycle because the commercial CaO powder did not have any anti-sintering resistant, such MgO as in CaMg-MOF, powder systems.

**Figure 4:**
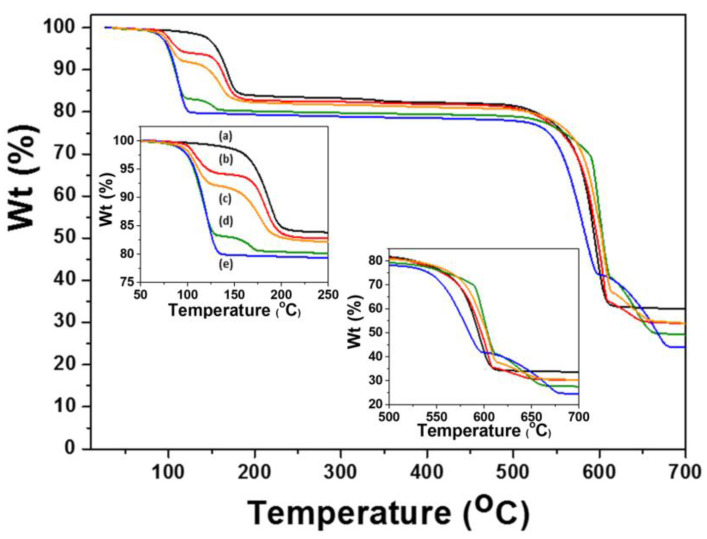
Thermal gravimetric analysis (TGA) curves of thermal property (**a**) Mg-MOF (**b**) Ca_0.6_-Mg_0.4_-MOF (**c**) Ca_0.97_-Mg_0.03_-MOF (**d**) Ca_0.97_-Mg_0.025_Al_0.005_-MOF (**e**) Ca-MOF.

The DTA of the samples were analyzed under continuous N_2_ gas flow with a heating rate of 5 °C /min. from room temperature up to 800 °C. The results in Figure 4 show that, in single metal component, the weight loss of Ca-MOF is 21 wt.% in the first step between 70 and 125 °C, the weight loss of Mg-MOF is 17% between 150 and 186 °C, mainly because of the removal of water molecules on the surface and coordinated ones. For multi-metal components, such as Ca_0.97_-Mg_0.03_-MOF and Ca_0.97_-Mg_0.025_Al_0.005_-MOF, the first part can be divided into two steps loss at 100 °C–120 °C and 125 °C –185 °C, indicating different coordination environment for water molecules to different metal ions. In the second part of weight loss step, Ca-MOF has 36.6 wt.% decreased between 510 °C to 600 °C, and Mg-MOF has 48.51 wt.% between 480 °C to 610 °C. For multi-metals Ca-Mg-MOFs, the weight losses are 43.6 wt.%–46.2 wt.%.

**Figure 5:**
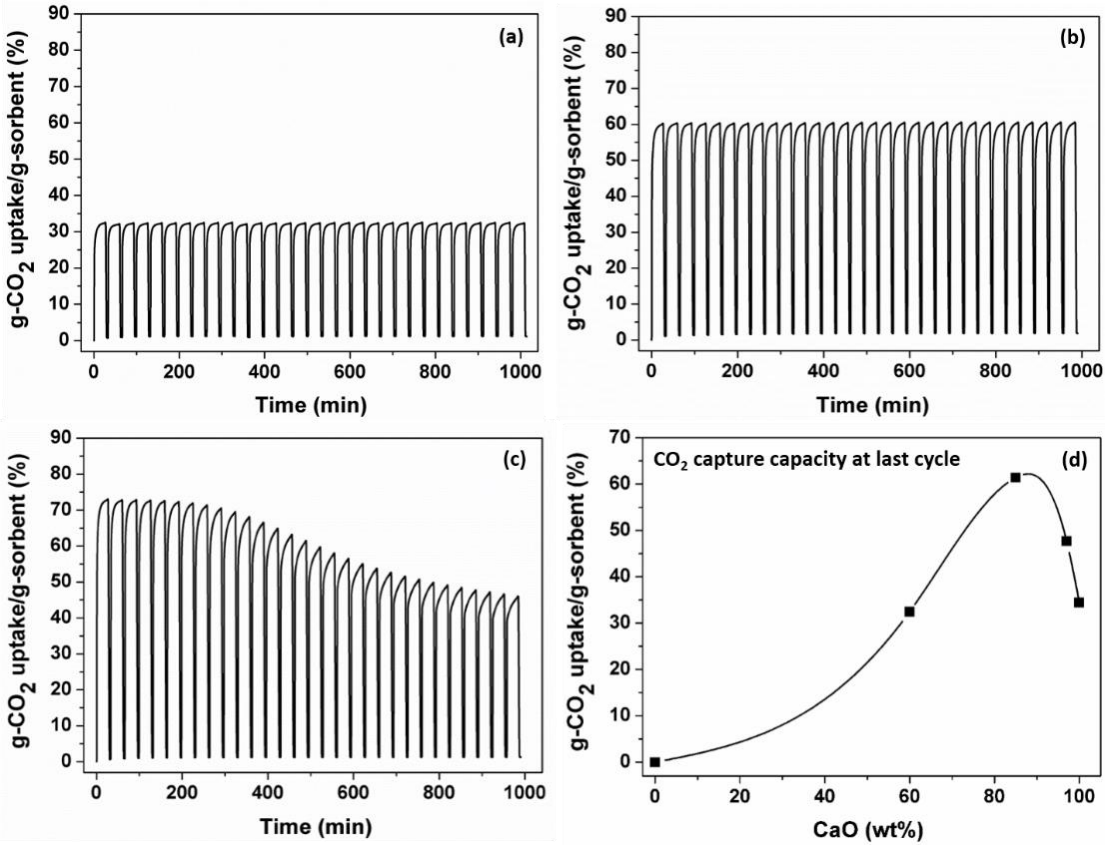
CO_2_-uptake of multiple carbonation–calcination cycles of (**a**) Ca_0.6_Mg_0.4_-MOF-O (**b**) Ca_0.85_Mg_0.15_-MOF-O (**c**) Ca_0.97_Mg_0.03_-MOF-O, and (**d**) the comparison of capture capacity above.

Figure 5 shows the results of multiple carbonation-calcination tests of CaMg-MOF-O with 30 cycles, which illustrated that both Ca_0.6_Mg_0.4_-MOF-O and Ca_0.85_Mg_0.15_-MOF-O not only have high CO_2_ capture amount of 33 wt.% and 61 wt.% but also demonstrate excellent long-term stability up to 94% and 96%, respectively, due to the existence of the anti-sintering component MgO. Since both MgO and CaO are rocksalt structure with six bonds to O for each Mg or Ca, meaning that at least 1/6 bond in Ca-O to react with Mg to form Ca-O-Mg in order to avoid the complete contact or full aggregation. Based on the concept, 0.15 mole MgO should be enough as anti-sintering resistant aid for avoiding the full aggregation of CaO or CaCO_3_ particles. That is the possible reason for the Ca_0.85_Mg_0.15_-MOF-O keeping long term stability with a higher CO_2_ capture content.

On the contrary, although Ca_0.97_Mg_0.03_-MOF-O with more CaO displayed the highest 72.82 wt.% capacity of CO_2_ capture, it can be maintained only up to the seventh cycle. After that, the capture decayed rapidly decayed to 43.89 wt.% on the 30th cycle, resulting in only 60% stability due to vigorous sintering and the particle growth effect. Figure 5d summarizes the CO_2_ capture capacity of the CaMg-MOF-O sorbent dependent on the CaO content at the last (30th) capture cycle, which indicates that there is also a maximum weight fraction of CaO (between 85 and 90%) in the sorbent, beyond which the sorbent loses its sintering-resistant MgO is not enough for reducing the aggregation during the multiple carbonation-calcination cycles.

### 3.2. Phase and Microstructure of Multi-Metals CaMgAl-MOFs

**Figure 6:**
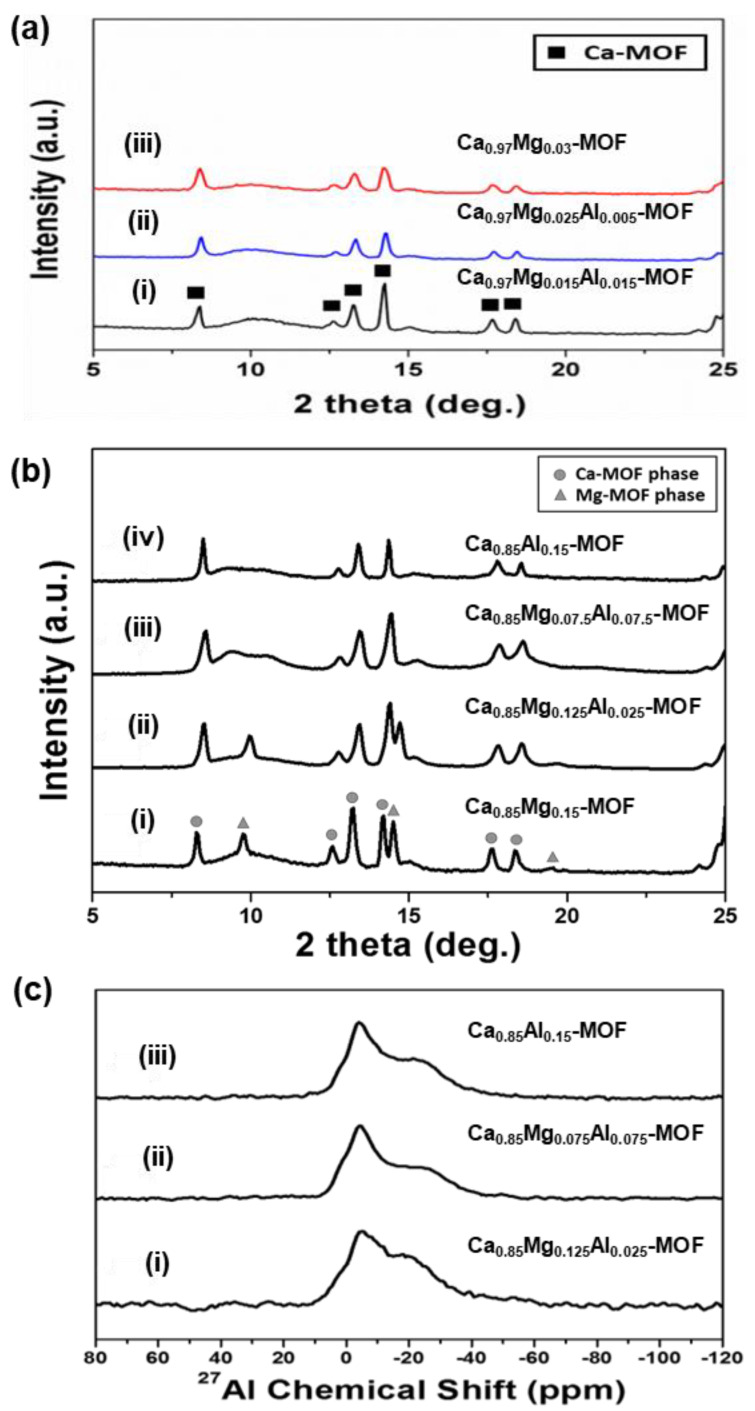
(**a**) XRD patterns of (i) Ca_0.97_Mg_0.03_-MOF, (ii) Ca_0.97_Mg_0.025_Al_0.005_-MOF, (iii) Ca_0.97_Mg_0.015_Al_0.015_-MOF. (**b**) XRD patterns of (i) Ca_0.85_Mg_0.15_-MOF, (ii) Ca_0.85_Mg_0.125_Al_0.025_-MOF, (iii) Ca_0.85_Mg_0.07.5_Al_0.07.5_-MOF, (iv) Ca_0.85_Al_0.15_-MOF. (**c**) ^27^Al NMR results of (i) Ca_0.85_Mg_0.125_Al_0.025_-MOF, (ii) Ca_0.85_Mg_0.075_Al_0.075_-MOF, (iii) Ca_0.85_Al_0.15_-MOF.

The abovementioned results showed that Ca_0.85_Mg_0.15_-MOF-O exhibited better cycling stability of CO_2_ capture when compared to Ca_0.97_Mg_0.03_-MOF-O but the latter has a higher CO_2_ capture capacity. Although MgO and Al_2_O_3_ have been known as anti-sintering agents against the carbonation-calcination cyclic degradation, the role of Mg-O and Al-O in CaMgAl MOFs on high temperature CO_2_ sorption are significantly different. Liu et al. studied the CO_2_ capture capacity of the CaO-MgO sorbents that were synthesized from calcium D-gluconate Monohydrate (CG) and magnesium D-gluconate hydrate (MG) and reported possible maximum weight fraction of CaO in the CaO-MgO sorbent in between 75 and 85% to maintain high stable performance over 24th capture cycle of utilization, which was attributed to MgO nanocrystalline that formed in the course of multistep decomposition of these precursors [22]. In our previous study, we found that the Al^3+^ cation in the calcined Ca-Al layered double hydroxide (Ca-Al-CO_3_ LDHs) can be incorporated into CaO matrix to form poor crystalline Ca-Al-O domains that can act as a physical barrier to separate the CaO nanoparticles. Therefore, in this study, multi-metal Ca_0.97_Mg_0.03_-MOF were selected for investigating the effect of partial Al substitution for Mg on the CO_2_ capture performance effect by keeping 3 mol% of the total Mg + Al amounts, which was denoted as Ca_0.97_Mg_0.025_Al_0.005_-MOF, and Ca_0.97_Mg_0.015_Al_0.015_-MOF. The PXRD of Ca_0.97_(Mg/Al)_0.03_-MOF system in Figure 6a presented Ca-MOF phase and a much broader and weaker peak at 2θ~10°, where, without obvious phase difference, can be observed from the XRD as changing the substitution of Al for Mg in this composition. Therefore, a higher Mg content of Ca_0.85_(Mg/Al)_0.15_-MOF was used to understand the phase evolution of partial Al substitution for Mg. Figure 6b showed that, with a third metal (Al) incorporating into the CaMg-MOF, the characteristic peaks of Ca-MOF(8.5°, 14.3°) and Mg-MOF phase (9.7°, 14.7°) were shifted to a higher 2 theta for both Ca_0.85_Mg_0.15_-MOF and Ca_0.85_Mg_0.125_Al_0.025_-MOF, indicating Al ion possibly dissolved in the Ca-MOF and Mg-MOF, which was mainly attributed to the smaller ionic size of Al^3+^ (~54 pm) than Ca^2+^ (~114 pm) and Mg^2+^(~86 pm). Here, it was noted that a further increasing Al substitution, the peak intensity of Mg-MOF phase almost disappeared and transformed into an unknown broad phase around 10° for Ca_0.85_Mg_0.07.5_Al_0.07.5_-MOF (Figure 6b-(iii)). In particular, when Mg was completely replaced by Al, Ca_0.85_Al_0.15_-MOF only displayed Ca-MOF phase with a weaker broad at 2 theta 8–12° in Figure 6b-(iv), indicating that Al ion can not form Al-MOF, but it was probably distributed in the MOF structure or existed in Al-containing amorphous phase. However, we further analyzed the powders with ^27^Al solid state NMR to assure the coordination environment of the Al in the structure since the PXRD results cannot provide the structure information about Al component. The results in Figure 5c shows that the signals located at −3.4 ppm indicated that Al element existed in an octahedral environment, six-coordinated with the COO^−^ groups, and H_2_O molecules. However, the broad band centered at −25 ppm represented a typical character of amorphous phase, which implied it might only have short-range order bonding [23]. This indicates that the Al^3+^ cations could be completely incorporated into the MOF framework, or may form an amorphous AlO_x_ domain that was not detectable by using XRD.

**Figure 7:**
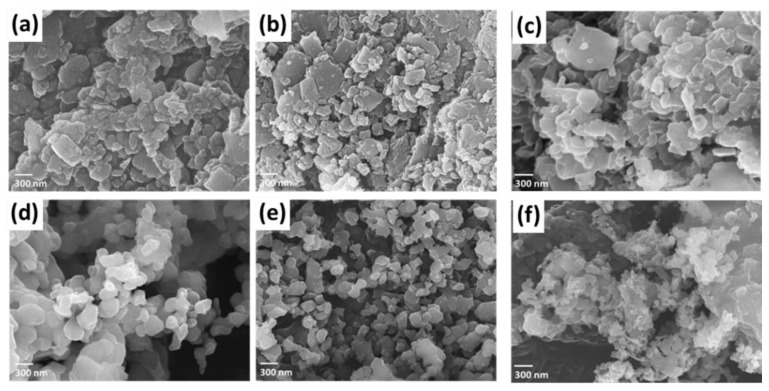
SEM images of (**a**) Ca0.97Mg0.03-MOF, (**b**) Ca0.97Mg0.025Al0.005-MOF, (**c**) Ca0.97Mg0.015Al0.015-MOF, (**d**) Ca0.97Mg0.03-MOF-O, (**e**) Ca0.97Mg0.025Al0.005-MOF-O, and (**f**) Ca0.97Mg0.015Al0.015-MOF-O.

It was found that only Ca-MOF and Mg-MOF can be formed in CaMgAl-MOF samples, meaning that the addition of Al to CaMg-MOF system would affect the nucleation and growth of Ca-MOF and Mg-MOF, according to Figure 6b. According to literature reports [24,25], the CO_2_ capture amount and cycle stability of calcium-based CaMgAl-MOF sample are more important and strongly related to particle size and aggregation. The morphology of partial Al substitution for Mg in Ca_0.97_Mg_0.03_-MOF system was further examined while using SEM. Without Al substitution, Ca_0.97_Mg_0.03_-MOF system was mainly composed of well-defined crystalline Ca-MOF with a rectangular shape around 200–700 nm in length, as shown in Figure 7a. However, as Al substituted for Mg in both Ca_0.97_Mg_0.03_-MOF and Ca_0.97_Mg_0.015_Al_0.015_-MOF systems, an obvious change in morphology and crystal size can be seen for Ca_0.97_Mg_0.025_Al_0.005_-MOF, indicating the nucleation and growth of Ca-MOF and Mg-MOF have been affected by Al substitution. They both presented a mixture morphology composed of large like-square smooth particles and irregular nanoparticles, as shown in Figure 7b,c. When the samples was treated at 600 °C to convert MOF precursors to form CaO-based oxide sorbents, The morphology of the heated samples can be further observed while using SEM in Figure 7d–f. Ca_0.97_Mg_0.03_-MOF-O and Ca_0.97_Mg_0.025_Al_0.005_-MOF-O both formed pebble-like crystals with uniformed particle size of around 200–300 nm for Ca_0.97_Mg_0.03_-MOF-O, and 150–200 nm for Ca_0.97_Mg_0.025_Al_0.005_-MOF-O in Figure 7d,e, respectively. However, Ca_0.97_Mg_0.025_Al_0.005_-MOF-O with minor Al substitution exhibited smaller particle with widely random distribution. On the contrary, Ca_0.97_Mg_0.015_Al_0.015_-MOF-O presented bigger irregular agglomerates and denser structure in Figure 7f, eventually resulting in a lower CO_2_ uptake.

### 3.3. Long-term CO_2_ Carbonation-Calcination Cyclic Performances of Al-Substitution Ca-Mg-MOF

**Figure 8:**
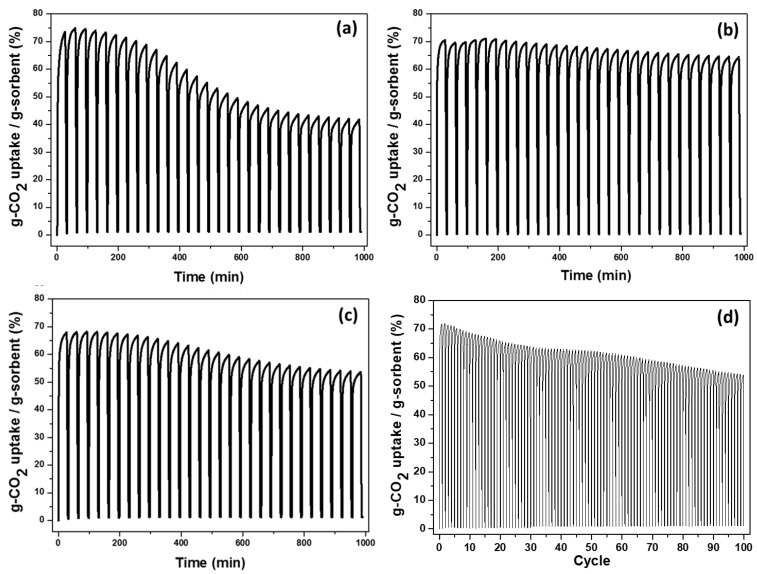
CO_2_-uptake of multiple carbonation-calcination cycles of (**a**) Ca_0.97_Mg_0.03_-MOF-O, (**b**) Ca_0.97_Mg_0.025_Al_0.005_-MOF-O, (**c**) Ca_0.97_Mg_0.015_Al_0.015_-MOF-O, and (**d**) CO_2_-uptake of 100 carbonation-calcination cycles of Ca_0.97_Mg_0.025_Al_0.005_-MOF-O.

**Figure 9:**
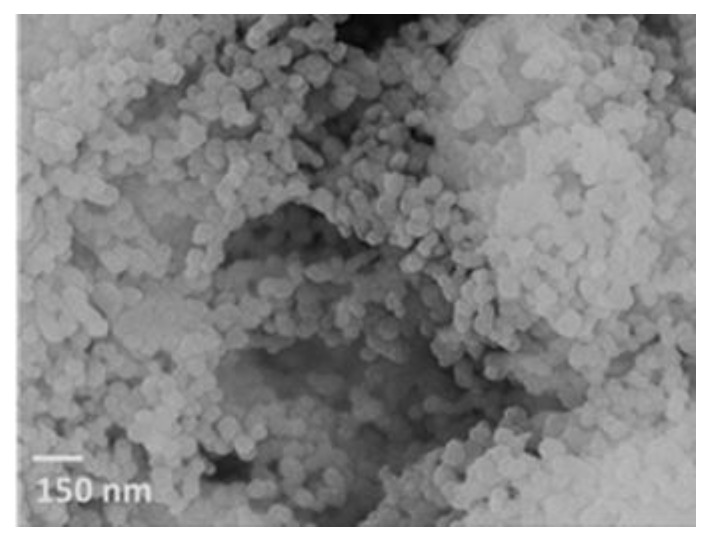
SEM images of Ca_0.97_Mg_0.025_Al_0.005_-MOF-O powder subjected to multiple carbonation-calcination for 100 cycles.

The Ca_0.97_Mg_0.03_-MOF-O with a higher CaO content presented a larger CO_2_ capture capacity when compared to the Ca_0.85_Mg_0.15_-MOF-O since the CaO adsorbent plays a more significant role in CO_2_ capture at high temperature. However, it was noted that the Ca_0.97_Mg_0.03_-MOF-O still displayed a cycle-degradation behavior. Long-term CO_2_ carbonation-calcination cyclic tests were performed for 30 cycles in order to further investigate the effect of partial Al substitution for Mg in the Ca_0.97_Mg_0.03_-MOF system on the CO_2_ capture character and cycle stability. As shown in Figure 8a, Ca_0.97_Mg_0.03_-MOF-O has the highest CO_2_ capacity about 74 wt.% at first cycle. However, there was a rapid decay after the fifth cycle and finally reduced to 40 wt.% with an overall 54.6% stability. With 0.5 mol% Al substituted for Mg in the Ca_0.97_Mg_0.03_-MOF-O structure in Figure 8b, Ca_0.97_Mg_0.025_Al_0.005_-MOF-O displayed the highest capacity about 70 wt.% in the first 10 cycles and maintained the CO_2_ capacity to 65 wt.% after 30 cycles with a well stability of 92% when compared to Ca_0.97_Mg_0.03_-MOF-O. Further, although further increasing Al substitution to 1.5 mole % still kept a high CO_2_ capture capacity (68 wt.%) with well stable up to 85% in the 30 cycles, the overall CO_2_ capture performance was affected, as shown in Figure 8c. The results in Figure 8b have demonstrated the excellent performance of Ca_0.97_Mg_0.025_Al_0.005_-MOF-O in the repeated carbonation-calcination cyclic test. The number of cycles was prolonged to one hundred cycles in order to explore the limit of the sorbent, as shown in Figure 8d. The highest capacity can be reached at the first cycle of 72 wt.% and, after the 30th cycle, the capacity was held at 65 wt.%, reaching a relatively stable structure to maintain 93% stability of original CO_2_ capture capacity. Subsequently, the capacity was again slowly decayed but at last it still has 55 wt.% capacity, resulting in an overall 77% stability at 100 cycle test. The SEM image presented in Figure 9 showed that after 100-cycle carbonation-calcination at temperature from RT to 700 °C was applied to the Ca_0.97_Mg_0.025_Al_0.005_-MOF-O sample, we found that particle aggregation was formed. However, the particle morphology and size of Ca_0.97_Mg_0.025_Al_0.005_-MOF-O sample were still unchanged with uniform distribution. These results demonstrated that, by using multi-metals CaMgAl-MOF as the precursor for CaO-based sorbent, and tuning the amount of Ca, Mg, and Al compositions, the Ca_0.97_Mg_0.025_Al_0.005_-MOF-O sorbent presented a significant enhancement in CO_2_ capture efficiency and cycle stability suitable for high temperature utilization.

## 4. Conclusions

In summary, we have successfully synthesized multi-metal MOFs, CaMgAl-MOFs as precursors to fabricate CaO-based sorbents for CO_2_ capture. The sorbents showed excellent CO_2_ capture performances and high resistance toward thermal sintering under severe conditions in the high temperature condition. By partially substituting Al for Mg, the CaMgAl-MOF-O series showed better stability than CaMg-MOF-O sorbents because the supported amorphous Al-O phase was formed in between. Furthermore, the Ca_0.97_Mg_0.025_Al_0.005_-MOF-O showed best optimization in CO_2_ capture (CaO) and cycle stability (due to the anti-sintering of MgO and Al_2_O_3_). An outstanding performance in 100 cyclic tests with the highest CO_2_ capacity of 71.8 wt.% and excellent stability of 77% can be achieved. The results may help future research of multi-metal MOFs and hopefully it can be further applied for the mitigation of CO_2_ emission.

## Figures and Tables

**Table 1:** Sample codes of CaMgAl-MOFs.

Composition (Mole %)	Ca	Mg	Al
Sample Code			
Ca_0.85_Mg_0.15_-MOF	85	15	0
Ca_0.85_Mg_0.125_Al_0.025_-MOF	85	12.5	2.5
Ca_0.85_Mg_0.075_Al_0.075_-MOF	85	7.5	7.5
Ca_0.85_Al_0.15_-MOF	85	0	15
Ca_0.97_Mg_0.03_-MOF	97	3	0
Ca_0.97_Mg_0.025_Al_0.005_-MOF	97	2.5	0.5
Ca_0.97_Mg_0.02_Al_0.01_-MOF	97	2	1
Ca_0.97_Mg_0.015_Al_0.015_-MOF	97	1.5	1.5
